# 2238. Estimating the impact of antibiotic exposure on antibiotic resistance in uncomplicated UTI using machine learning causal inference

**DOI:** 10.1093/ofid/ofac492.1856

**Published:** 2022-12-15

**Authors:** Sanjat Kanjilal, Hyewon Jeong, Yidan Ma, Alex Wei, Kexin Yang, David Sontag

**Affiliations:** Department of Population Medicine, Harvard Medical School and Harvard Pilgrim Healthcare Institute, Boston, Massachusetts; Computer Science and Artificial Intelligence Laboratory, Massachusetts Institute of Technology, Cambridge, Massachusetts; Department of Biostatistics, Harvard School of Public Health, Boston, Massachusetts; Department of Biostatistics, Harvard School of Public Health, Boston, Massachusetts; Department of Biostatistics, Harvard School of Public Health, Boston, Massachusetts; Computer Science and Artificial Intelligence Laboratory, Massachusetts Institute of Technology, Cambridge, Massachusetts

## Abstract

**Background:**

Incorporating an antibiotic’s propensity for engendering resistance to itself and other antibiotics is a potentially useful strategy for preventing antimicrobial resistance (AMR), but prospective studies have been difficult to generalize to outpatients and retrospective studies are prone to design errors and model misspecification. To address this gap, we apply causal inference with targeted maximum likelihood estimation (TMLE) using machine learning, to data from the electronic health record to define the antibiotic use-resistance relationship for common outpatient therapies used to treat urinary tract infection (UTI).

**Methods:**

We estimated the risk of AMR in response to treatment in a cohort of outpatients with uncomplicated UTI in the Mass General Brigham health system between 2016 and 2021. We sought to emulate a randomized controlled trial using the targeted maximum likelihood (TMLE) approach with logistic regression, random forests, multilayer perceptrons, and XGBoost to mitigate confounding by indication and to model the outcome (Figure 1). Potential confounders include demographics, comorbidities and prior microbiology, windowed in time for temporally varying features (Figure 2). We quantified the average treatment effect (ATE) of exposure to nitrofurantoin (NIT, target trial 1) or fluoroquinolones (FQs, target trial 2) to any other antibiotic type on the risk of AMR to NIT, FQs or amoxicillin-clavulanate at 12 months post-exposure.

**Figure 1:**
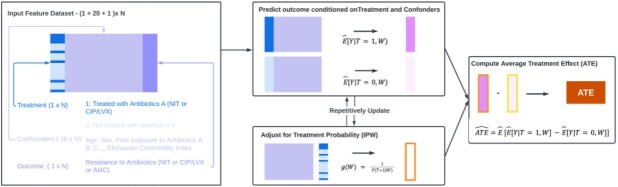
Analytic framework

**Figure 2:**
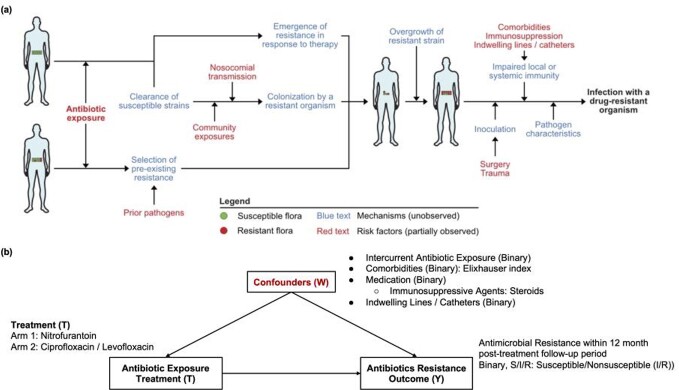
Causal diagram

**Results:**

Our final cohort consisted of 4,573 patients with no baseline AMR or antibiotic exposure in the previous 12 months who were treated with NIT, FQs or oral beta-lactams. XGBoost models significantly outperformed other model types. Compared to other antibiotics, the ATE of NIT exposure to NIT resistance at 12 months was 0.05 (0.04 – 0.07) and for FQ resistance was 0.06 (0.05, 0.08). Exposure to NIT had no impact on the risk of resistance to AMC at 12 months. Exposure to FQs had no impact on resistance to FQs, NIT or AMC at 12 months (Figure 3).

**Figure 3:**
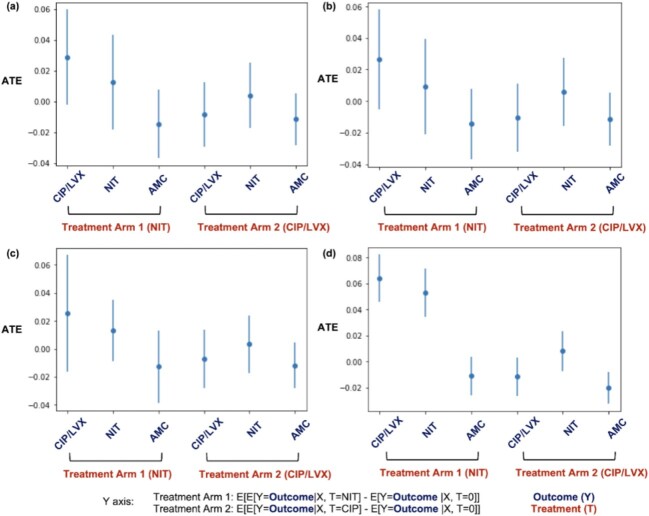
Average treatment effects

**Conclusion:**

Outpatients treated with NIT had a higher risk of AMR at 12 months than those treated with FQs. Future work will focus on including hospital exposures and immunosuppression into models and infer impact using a wider range of treatments.

**Disclosures:**

**Sanjat Kanjilal, MD, MPH**, GlaxoSmithKline: Advisor/Consultant|Roche Diagnostics: Honoraria **David Sontag, PhD**, Adobe: Grant/Research Support|ASAPP: Advisor/Consultant|Cureai Health: Stocks/Bonds|Facebook: Grant/Research Support|Google: Grant/Research Support|IBM: Grant/Research Support|SAP: Grant/Research Support|Takeda: Grant/Research Support.

**Figure 1:** Analytic framework

Framework for targeted maximum likelihood estimation (TMLE) of causal impacts.

**Figure 2:** Causal diagram

a) Conceptual model for the emergence of AMR with observed and unobserved features and b) causal diagram for inference model.

**Figure 3:** Average treatment effects

ATEs for the impact of NIT or FQs on the risk of AMR to NIT, FQs or AMC at 12 months using a) logistic regression, b) random forest, c) multilayer perceptron and d) XGBoost models

